# Feasibility of Deploying Inhaler Sensors to Identify the Impacts of Environmental Triggers and Built Environment Factors on Asthma Short-Acting Bronchodilator Use

**DOI:** 10.1289/EHP266

**Published:** 2016-06-24

**Authors:** Jason G. Su, Meredith A. Barrett, Kelly Henderson, Olivier Humblet, Ted Smith, James W. Sublett, LaQuandra Nesbitt, Chris Hogg, David Van Sickle, James L. Sublett

**Affiliations:** 1 Division of Environmental Health Sciences, School of Public Health, University of California, Berkeley, Berkeley, California, USA; 2 Propeller Health, San Francisco, USA; 3 Office of Civic Innovation, Louisville Metro Government, Louisville, USA; 4 Family Allergy and Asthma, Louisville, USA; 5 District of Columbia Department of Health, Washington, DC, USA; 6 University of Wisconsin School of Medicine and Public Health, Madison, Wisconsin, USA

## Abstract

**Background:**

Epidemiological asthma research has relied upon self-reported symptoms or healthcare utilization data, and used the residential address as the primary location for exposure. These data sources can be temporally limited, spatially aggregated, subjective, and burdensome for the patient to collect.

**Objectives:**

First, we aimed to test the feasibility of collecting rescue inhaler use data in space–time using electronic sensors. Second, we aimed to evaluate whether these data have the potential to identify environmental triggers and built environment factors associated with rescue inhaler use and to determine whether these findings would be consistent with the existing literature.

**Methods:**

We utilized zero-truncated negative binomial models to identify triggers associated with inhaler use, and implemented three sensitivity analyses to validate our findings.

**Results:**

Electronic sensors fitted on metered dose inhalers tracked 5,660 rescue inhaler use events in space and time for 140 participants from 13 June 2012 to 28 February 2014. We found that the inhaler sensors were feasible in passively collecting objective rescue inhaler use data. We identified several environmental triggers with a positive and significant association with inhaler use, including: AQI, PM10, weed pollen, and mold. Conversely, the spatial distribution of tree cover demonstrated a negative and significant association with inhaler use.

**Conclusions:**

Utilizing a sensor to capture the signal of rescue inhaler use in space–time offered a passive and objective signal of asthma activity. This approach enabled detailed analyses to identify environmental triggers and built environment factors that are associated with asthma symptoms beyond the residential address. The application of these new technologies has the potential to improve our surveillance and understanding of asthma.

**Citation:**

Su JG, Barrett MA, Henderson K, Humblet O, Smith T, Sublett JW, Nesbitt L, Hogg C, Van Sickle D, Sublett JL. 2017. Feasibility of deploying inhaler sensors to identify the impacts of environmental triggers and built environment factors on asthma short-acting bronchodilator use. Environ Health Perspect 125:254–261; http://dx.doi.org/10.1289/EHP266

## Introduction

Despite efforts to address the substantial health and economic burden of asthma in the United States and around the world, it remains a significant health issue. Previous epidemiological studies trying to identify environmental triggers of asthma have relied upon aggregated and infrequently-reported asthma outcome measures, such as emergency department visits or hospitalizations, which lack temporal and spatial resolution due to aggregation to an annual basis and grouping to a ZIP code or county level. Other studies have used patient self-reported data to assess the location and frequency of symptoms; however, patient diaries have been demonstrated to be fraught with missing data, errors and are burdensome for the patient (Jordan et al. 2006; Rockenbauer et al. 2001; de Marco et al. 2002). Studies have also utilized a patient’s residential address as the primary location of exposure; however, exposure to known indoor and outdoor asthma triggers can occur in the community, at work, at home, at school, and elsewhere; therefore, residential address does not capture the full signature of exposure. Additionally, impacts from built environment factors other than the home cannot be effectively assessed. These limitations make it challenging to identify when and where asthma symptoms occur, and how personal environmental exposures might influence the pattern of symptoms (Guarnieri and Balmes 2014).

Patients with inadequately controlled asthma are at particularly high risk of exacerbations, hospitalization, and death, and they often have severely impaired quality of life (Peters et al. 2006). Asthmatic patients often use short-acting bronchodilators (or “rescue” inhalers) to gain relief from acute symptoms such as cough, wheeze or shortness of breath. Electronic sensors fitted onto rescue inhalers, which can capture the time and location of use, offer an immediate, objective signal of asthma activity and potential exposure and can contribute to public health surveillance and research (Nilsen et al. 2012; Barrett et al. 2013; Kumar et al. 2013).

This feasibility study had three distinct objectives. First, we aimed to evaluate the feasibility of collecting rescue inhaler use data in space–time using inhaler sensors. Second, we aimed to evaluate whether these collected data could identify environmental triggers and built environment factors associated with rescue inhaler use, and whether these findings would be consistent with the existing literature. These two objectives were the primary focus of this paper. Third, the study aimed to assess the feasibility of using inhaler sensors and a mobile health platform to improve asthma outcomes for participants, such as the frequency of rescue inhaler use and symptom-free days. These clinical objectives and results are discussed elsewhere and will not be the focus of this paper (Van Sickle et al. 2015).

## Methods

### Study Site

The city of Louisville, located in Jefferson County, Kentucky, ranks among the top 20 “most challenging places to live with asthma” in the United States (Asthma and Allergy Foundation of America 2013). Asthma surveillance activities driven by the local public health department must rely upon asthma hospitalization records and national survey prevalence data, which are aggregated at a ZIP-code level and are often more than a year old. In order to address these limited surveillance data and better understand local environmental drivers of asthma, municipal leaders in Louisville formed a public-private collaboration to improve asthma surveillance and inform policy decision-making (AIR Louisville 2015).

### Participant Enrollment

The program recruited participants through convenience sampling from community events, clinics and retail pharmacies in Jefferson County. Participants were eligible if they reported a physician diagnosis of asthma and had a current prescription for a compatible inhaled short-acting bronchodilator medication. Participants were excluded if they *a*) were under the age of 4; *b*) did not speak English; *c*) did not have access to the internet; or *d*) had substantial respiratory co-morbidity such as chronic obstructive pulmonary disease. The study was reviewed and approved by the Copernicus Group Independent Review Board, and received written informed consent from all participants, including written consent from guardians on behalf of children under 18. Demographic data were collected via surveys. The parents of children < 12 years old assisted with completing the survey. In general, the parents of children < 18 years old were also trained on the sensors and mobile health platform.

### Collection of Rescue Inhaler Use in Space and Time

Rescue inhaler use events were captured using a wireless inhaler sensor (Propeller Health, Madison, WI). The Propeller sensor and platform comprise a U.S. Food and Drug Administration (FDA) cleared digital therapeutic that combines inhaler sensors, mobile applications, and predictive analytics for patients and clinicians (Van Sickle et al. 2013). The sensor is compatible with the majority of available metered dose inhalers, and it objectively records their use, capturing the date, time, and number of actuations. The sensor transmits the information via Bluetooth to a paired smartphone, which then records the geographic location of the use event. Participants without smartphones were not excluded from the study. Instead, they utilized a wireless hub, which transmits actuation data but does not enable the capture of global positioning system (GPS) locations. The smartphone and hubs securely upload inhaler use data to encrypted servers. Following American Thoracic Society/European Respiratory Society guidance, actuations occurring within a 2 min time period were considered a single rescue inhaler use event (Reddel et al. 2009).

The digital health platform enables a participant to self-report information on perceived symptoms and triggers associated with a rescue inhaler use event, and report whether the inhaler was used pre-emptively. Before our analysis, we removed all rescue inhaler events marked as pre-emptive.

The Propeller Health System is considered an electronic Metered Dose Inhaler (MDI) Accessory according to the Code of Federal Regulations (FDA 2016) and required a 510(k) clearance reviewed by the FDA’s Center for Devices and Radiological Health (CDRH). Performance bench testing of sensor actuations and data capture demonstrated reliable functionality according to applicable standards and testing, including Federal Communications Commission (FCC) licensing and wireless Bluetooth technology (FDA 2013).

### Measurements of Environmental Triggers

**Figure 1:**
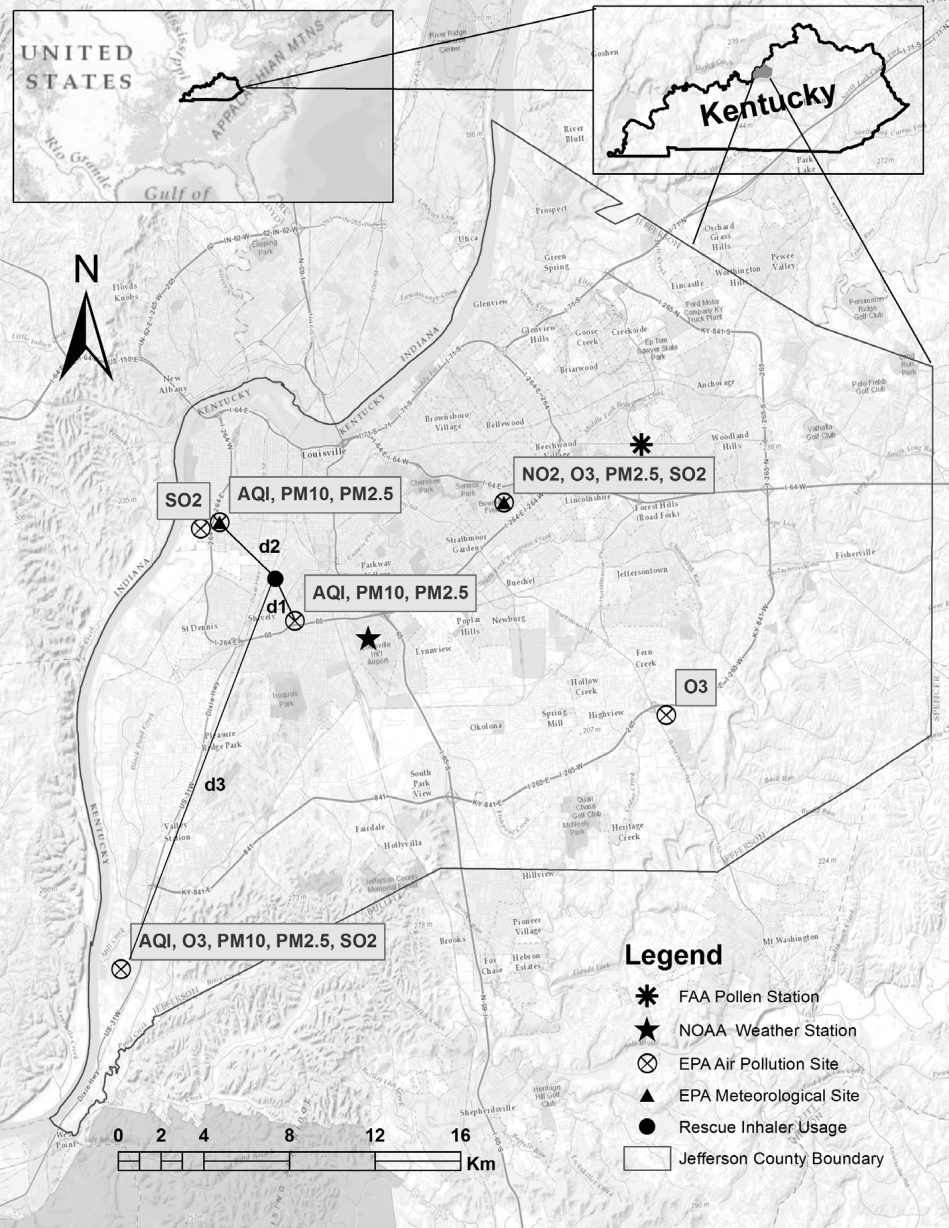
The relative location of Jefferson County, Kentucky, and its air pollution, pollen and weather monitoring sites. For the rescue inhaler use location marked on the map (solid circle), the estimation of PM_10_ concentration as applied in Equation 3 can be obtained using the following method:

Environmental triggers assessed in this study included air pollutants, pollen, mold and meteorological factors. We acquired air pollutant data from the U.S. Environmental Protection Agency’s (EPA) Air Quality System (AQS) for the following criteria pollutants: nitrogen dioxide (NO_2_), ozone (O_3_), sulfur dioxide (SO_2_), and particulate matter with aerodynamic diameter ≤ 2.5 μm (PM_2.5_) and ≤ 10 μm (PM_10_). We also collected Air Quality Index (AQI) data from the available monitoring stations (Figure 1). AQI is an index of daily ambient concentrations of up to five criteria air pollutants (i.e., O_3_, PM, carbon monoxide, SO_2_, and NO_2_). It is a piecewise linear function of a pollutant concentration, and it ranges from < 50 (good air quality) to over > 400 (poor air quality). If multiple pollutants are measured at a monitoring site, then the highest pollutant level is reported for AQI at that location (U.S. EPA 2013). The AQS pollutant data are collected at different temporal resolutions, including hourly concentrations for NO_2_ and SO_2_, daily concentrations for PM_2.5_ and PM_10_, a daily mean of 8 hr maximum for O_3_, and mean daily values for AQI. We used an inverse distance-weighting (IDW) algorithm to estimate pollutant concentrations for locations of rescue inhaler use per hour or per day using the AQS monitoring data (Figure 1). The spatially interpolated daily values were used to represent hourly measures of pollutants when only daily data were available. The concentration of a pollutant at location *j* of rescue inhaler use during hour *t* (*c_jt_*) was calculated using all known monitoring sites (*i* = 1, 2, …, n) concentration measurements (*c_ijt_*) (Equation 1):

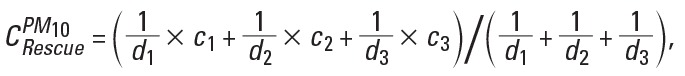

where *d_i_* and *c_i_* are respectively the distance to and concentration at the *i*th monitoring site. A hypothetical example is used here to demonstrate how we estimated exposure at a rescue inhaler use location through the inverse distance weighting algorithm. The figure was created by the authors of this paper using ArcGIS software (V9.3; Environmental Systems Research Institute, Redland, CA).

*c_jt_* = Σ*_i_* (*w_ij_* × *c_ijt_*)/Σ*w_ij_,* [1]

where *w_ij_* = 1/*d_ij_* and *d_ij_* is the distance between known monitoring station *i* and rescue inhaler use location *j*. For rescue inhaler use events without location data, daily or hourly regional mean statistics were used.

The pollen data for the study period were collected in Louisville by the staff at Family Allergy & Asthma from a monitoring station on the roof of the clinic (Figure 1), and included daily counts for mold spores, and tree, grass, and weed pollen. Because we were limited to daily data from just one monitoring location, all the inhaler-use events on a specific day were assigned the same pollen and mold counts.

We downloaded daily meteorological data, including wind speed, relative humidity, temperature, and atmospheric pressure, from U.S. EPA AQS sites (Figure 1). The IDW algorithm assigned daily meteorological conditions for the locations of rescue inhaler use. We also acquired daily precipitation, snow, and wind direction data from the National Oceanic and Atmospheric Administration (NOAA) for the Louisville International Airport (Figure 1). Wind direction data (0–360°) were reclassified into eight categories: north, northeast, east, southeast, south, southwest, west and northwest.

### Measurements of Built Environment Factors

We identified built environment factors that might influence rescue inhaler use, such as land use, land cover, and property characteristics. We acquired land use and property data from the Louisville/Jefferson County Information Consortium for 2014. For each rescue inhaler use location, corresponding land use characteristics (e.g., % residential) were calculated within a 250-m buffer, an area representing local influence on an individual (Su et al. 2011).

We also acquired land cover data from the National Land Cover Database for 2011 at a spatial resolution of 30 m. The land cover classes for vegetation included forest (deciduous, evergreen, and mixed), shrub land, and grassland/herbaceous cover. Our primary interest in including land cover was to examine the potential protective (e.g., reductions of air pollution by tree) or causal (e.g., pollen generation by weed) effect of vegetation on rescue inhaler use. Pollen counts were measured at the regional level and were temporally resolved (daily), while land cover vegetation classification data were spatially resolved using the 250-m buffer.

### Statistical Modeling

We first explored bivariate relationships between daily number of rescue inhaler use events and corresponding daily pollutant concentrations and meteorological conditions with time series plots and scatterplots. To be consistent with subsequent statistical modeling, we used the ratio of rescue inhaler use events each day to the number of active participants on that day. We then assessed the association of environmental triggers and built environment factors with inhaler use by implementing zero-truncated negative binomial models, and validating these results using three sensitivity analyses, which we describe in further detail.

### Detecting Environmental Associations With Rescue Inhaler Use

First, we identified the environmental triggers that might have an impact on rescue inhaler use through an unadjusted zero-truncated negative binomial model. Our goal was to model the number of events of rescue inhaler use of all active participants per day using average environmental exposures within the same day (Equation 2):

log(E (*Y_i_*)) = β_0_ + β_1_ × *X_i_* + log(*A_i_*) + ε*_i_*, [2]

where E (*Y_i_*) and *X_i_* are respectively the expected number of inhaler use events and the environmental predictor at the *i*th day. Negative binomial models were applied because initial data exploration identified that the data for rescue inhaler use were highly overdispersed, with the variance about 10 times the mean. We used rate ratios (RR) in interquartile range (IQR) increments and corresponding 95% confidence intervals (CI) to identify the degree of impact of environmental triggers on rescue inhaler use. RR is a relative difference measure used to compare the incidence rates of events. For environmental triggers that were spatially resolved, a single mean statistic averaged from all the locations of rescue inhaler use each day was used. *A_i_* is the total number of active participants at the *i*th day, representing the participants who were tracking their inhaler use on that day. An active participant was defined as having a sensor that was on and capable of transmitting data, however not all active participants used their rescue inhaler on any given day. We used the number of active participants each day as an offset to adjust for bias. ε*_i_* is the model error term.

Second, we expanded the unadjusted zero-truncated negative binomial models to include multiple environmental factors that might influence inhaler use simultaneously, including air pollution, pollen, and meteorological data (Equation 3):

log(E (*Y_i_*)) = β_0_ + β_1_ × *X*_1_ *_i_* + β_2_ × *X*_2_ *_i_* + β_3_ × *X*_3_ *_i_* + log(*A_i_*) + ε*_i_*, [3]

where X_1_ *_i_*, X_2_ *_i_*, and X_3_ *_i_* are, respectively, vectors of air pollution, pollen, and mold spore counts, and meteorological information for the *i*th day. The meteorological information included wind speed, precipitation, temperature, and season. We used a polynomial function for the impact of temperature. Again, rate ratios in IQR increments and 95% CI were used to represent the relative impacts of environmental triggers on rescue inhaler use. Due to the collinearity between air pollutants, we developed separate models for AQI and each pollutant.

For the environmental trigger models, we assessed the “immediate” effect of environmental exposure before a rescue inhaler event occurred as well as the effect of time-lagged exposures 1–3 days preceding the rescue inhaler event. Immediate effects were defined as occurring within the hour preceding an inhaler event (e.g., using pollutant concentrations at 1000 hours for a 1035 hours event). We used each discrete rescue inhaler event’s time and location to determine the environmental exposure. For pollutants with hourly data available, the concentrations at the specific space–time for which rescue inhaler use occurred were assigned. For pollutants with only daily data available, the interpolated daily concentrations at the locations of rescue inhaler use were used. In our zero-truncated negative binomial modeling process, the exposure values from all rescue inhaler events occurring in a single day were averaged to daily means. For assessing time-lagged exposures, lagged exposures were calculated by averaging concentrations measured from all the locations of rescue inhaler use that occurred on the respective preceding 1–3 days.

### Detecting Built Environment Associations with Rescue Inhaler Use

We included property and land cover data in the models to identify the impacts of built environment characteristics, such as transportation infrastructure, land use or vegetation, on rescue inhaler use. We used the subset of rescue inhaler use data with geographic location data assigned to each event. A location of inhaler use at a specific time and day was linked to the land cover data based on the latitude and longitude information recorded by participants’ smartphones. Similar to the identification of environmental triggers of rescue inhaler use, we built unadjusted models to assess associations of rescue inhaler use with property types and land cover characteristics using Equation 2.

### Sensitivity Analyses

We tested the reliability of our findings by implementing three sensitivity analyses. To test the feasibility of using the individual observation as the unit of analysis, we used a subgroup of 80 participants for which we had demographic data and conducted two sensitivity analyses to identify associations of environmental triggers with rescue inhaler use. For each participant, we tracked their start date, end date, and the intervening active days the participant was in the program. First, we developed a generalized linear mixed model with repeated measures. For those active days on which an individual experienced a rescue inhaler use event (response = 1), we extracted the corresponding environmental exposure values based on the time and location of the event. If location data were not available, regional mean statistics at the time of exposure were applied. For those active days on which an individual did not experience a rescue inhaler use event (response = 0), we extracted the corresponding regional mean exposure statistics for each day. We tried to control for person-level confounding by including race/ethnicity, sex, smoking status, and pet ownership. Studies show that race/ethnicity and smoking status are significantly correlated with disease severity and medication adherence and could be treated as a proxy for such measures (Boudreaux et al. 2003; Bitsko et al. 2014; Crocker et al. 2009; Wells et al. 2008). The second sensitivity analysis evaluated the impact of time-lagged exposures (up to 3 days preceding an event) for each of the 80 participants through similar repeated regression models. Time-lagged exposures were estimated using corresponding regional mean exposure statistics for the lagged days. We also conducted a third sensitivity analysis, which implemented the zero-truncated negative binomial regression models using only the inhaler use data that had geolocation information.

## Results

### Feasibility of Collecting Rescue Inhaler Use in Space–Time

We recruited 140 volunteer participants across Jefferson County who passively recorded their rescue inhaler use from 13 June 2012 to 28 February 2014. Of the 140 participants, 80 of them provided demographic information via surveys. Participants self-reported their race/ethnicity as African American (31.3%), Non-Hispanic White (57.5%), Hispanic (1.3%), Native American (2.5%) or other (7.5%) (Table 1). Among those 80 participants, 62.5% were female. We examined smartphone device ownership and access among participants: 27.1% used an iPhone, while 37.8% used an Android phone, with Samsung (17.1%), High Tech Computer Corporation (HTC) (15.0%) and Motorola (5.7%) ranking as the most popular devices. 35.1% of participants did not own a smartphone and used an alternative device or a wireless hub to transmit their data.

**Table 1:** The descriptive statistics of a subset of the participants and the phone types of all the participants enrolled in the feasibility study.

Participant characteristics	*n* (%)
Sex^*a*^
Male	30 (37.5)
Female	50 (62.5)
Race/ethnicity^*a*^
Black	25 (31.3)
White	46 (57.5)
Hispanic	1 (1.3)
Native	2 (2.5)
Other	6 (7.5)
Smoking status^*a*^
Smokers	21 (26.3)
Nonsmokers	59 (73.7)
Pet ownership^*a*^
With pets	42 (52.5)
Without pets	38 (47.5)
Phone type^*b*^
Non-smartphone/hub	49 (35.1)
HTC	21 (15.0)
Motorola	8 (5.7)
Samsung	24 (17.1)
iPhone	38 (27.1)
Note: HTC, High Tech Computer Corporation.^***a***^The statistics are for the 80 participants who submitted answers to initial surveys. ^***b***^Phone types are statistics for the 140 patients.	

**Figure 2:**
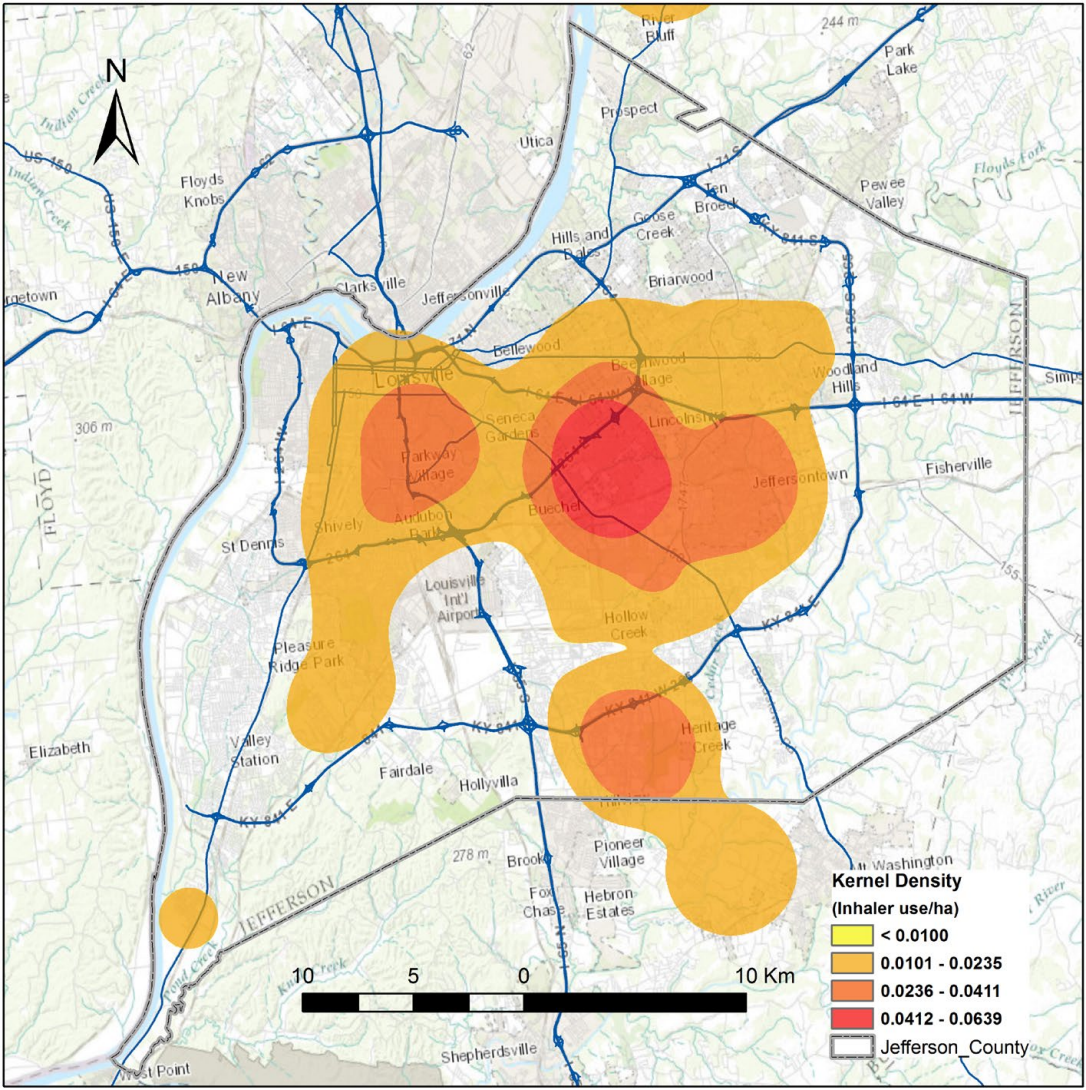
Hotspots of rescue inhaler use events in Jefferson County, Kentucky, generated using a kernel density function based on the locations where rescue inhaler use events occurred from June 2012 to February 2014. The figure was created by the authors of this paper using ArcGIS software (version 9.3; Environmental Systems Research Institute, Redland, CA).

The inhaler sensors collected a total of 10,475 actuations, which were grouped into 5,660 unique rescue inhaler use events. Of the rescue inhaler events logged, 100% successfully recorded information on the date, time, and the number of actuations. A subset of the inhaler use events were assigned GPS locations (23%), which enabled a kernel density analysis of the geographic distribution of the rescue inhaler use events (Figure 2). Of all participants, 35.1% did not own a smartphone and transmitted data via an alternate device or wireless hub, which are not capable of collecting GPS locations. We explored the patterns of geographic data capture across sex, race/ethnicity, time of day, and type of smartphone transmission device. We did not find any significant differences in GPS data capture for men vs. women, for race/ethnicity, for time of day (day 0600–2000 hours vs. night) or for type of smartphone.

### Detecting Environmental Associations with Rescue Inhaler Use

We first assessed the correlation coefficients (*r*) between environmental triggers and found that air pollutants had *r* values between 0.28 and 1.00, while their correlations with pollens from grass, tree, weed, and mold were < 0.20 (see Table S1). AQI was heavily impacted by PM_10_ (*r* = 1.0) and PM_2.5_ (*r* = 0.75). We overlaid the daily number of rescue inhaler use events with daily pollutant concentrations and temperature in time series plots (see Figures S1–S3), and explored the relationship between rescue inhaler use and temperature (see Figure S4).

We also identified the feasibility of using sensor-collected data to detect possible associations with environmental triggers, including air pollution, pollen, and mold, in the unadjusted zero-truncated negative binomial models (see Table S2). All the modeling results are expressed as RR (95% CI) through corresponding IQR increments. We found that AQI [1.201 (95% CI: 1.156, 1.248)], PM_10_ [1.204 (95% CI: 1.158, 1.251)], and NO_2_ [1.033 (95% CI: 1.000, 1.066)] were positively associated with inhaler use, but O_3_ had a negative association [0.913 (95% CI: 0.878, 0.950)] for immediate exposures, all significant at the 0.05 level. We also found that weed pollen [1.005 (95% CI: 1.004, 1.006)] and mold [1.153 (95% CI: 1.104, 1.203)] had positive associations but tree pollen [0.982 (95% CI: 0.977, 0.987)] and grass pollen [0.992 (95% CI: 0,985, 0.998)] had negative associations. In addition, we identified that the impacts of time-lagged exposures were similar to the immediate impact of daily exposures for all variables (see Table S2).

Next we built an adjusted environmental trigger model that also included meteorological variables (see Table S2). After including these additional environmental factors, AQI [1.055 (95% CI: 1.003, 1.108)] and PM_10_ [1.054 (95% CI: 1.002, 1.109)] were still positively and significantly associated with rescue inhaler use. O_3_ had a positive association for lag exposures of 1 day [1.082 (95% CI: 1.021, 1.146)] and 2 days [1.073 (95% CI: 1.012, 1.136)]. Tree pollen maintained a negative association [0.991 (95% CI: 0.985, 0.998)], but grass pollen [1.012 (95% CI: 1.004, 1.019)] and weed pollen [1.005 (95% CI: 1.004, 1.007)] demonstrated a positive association.

The three sensitivity analyses demonstrated associations that largely aligned with the zero-truncated negative binomial models. In the first sensitivity analysis (subgroup of 80 participants with demographic data), after controlling for race/ethnicity, sex, smoking status and pet ownership, AQI, and PM_10_ were found to maintain their positive and significant association with rescue inhaler use (see Table S3). Tree pollen maintained a negative association while weed pollen and mold maintained a positive association, all significant. In the second sensitivity analysis (time-lagged exposures), these relationships were also maintained (see Table S3). In the third sensitivity analysis (restricted to only geographically-defined data), the results were also largely maintained, except that the association with O_3_ was positive and significant, which was opposite of the result from the original analysis (see Table S4).

To summarize the consistent results from the two primary analyses and three sensitivity analyses, AQI, PM_10_, and weed pollen were found to be positively and significantly associated with rescue inhaler use in all five models. Mold was found to be positively and significantly association with inhaler use in four of the five models. Additionally, tree pollen demonstrated a negative and significant association in all five models.

### Detecting Built Environment Associations with Rescue Inhaler Use

In the unadjusted models of built environment factors (see Table S5), we found that vegetation cover [0.829 (95% CI: 0.800, 0.857)], which included trees, herbaceous and shrubland, was negatively associated with rescue inhaler use. When examined individually, tree cover [0.825 (95% CI: 0.796, 0.854)] and herbaceous cover [0.901 (95% CI: 0.872, 0.929)] were negatively associated with rescue inhaler use, but shrubland [0.993 (95% CI: 0.955, 1.025)] exhibited no significant associations.

We also found that industrial land use [0.992 (95% CI: 0.987, 0.996)] and residential land use categories [0.880 (95% CI: 0.829, 0.933)] had significant and negative associations, while public utilities [1.009 (95% CI: 1.005, 1.013)] and exempt land use categories [1.153 (95% CI: 1.113, 1.194)] had positive and significant associations. Public utilities included areas such as railroads, pipelines, electricity delivery, and other utilities. Exempt properties include those owned by communities, churches, hospitals, colleges, cities, counties, state or federal authorities. Upon further investigation into the detailed exempt properties, we found that educational institutions [1.008 (95% CI: 1.002, 1.013)], religious gathering places [1.094 (95% CI: 1.068, 1.121)], residential condominium master lots (e.g., a central courtyard surrounded by the residential buildings) [1.014 (95% CI: 1.009, 1.020)], and metro government sites [1.054 (95% CI: 1.045, 1.063)] were all significantly and positively associated with rescue inhaler use.

## Discussion and Conclusion

### Feasibility of Collecting Inhaler Use Data

In this study, we tested the feasibility of using inhaler sensors to collect rescue inhaler use data in space–time, and to use these data to detect environmental triggers and built environment factors associated with asthma symptoms. We found that the sensors are reliable in collecting medication use data: 100% of the 10,475 rescue inhaler actuations successfully recorded the date and time. Geographic data were not captured on all events primarily because participants without smartphones used alternative devices or wireless hubs (35.1%), which are not capable of collecting GPS data. It was known before the study started that these participants would not be collecting GPS data, but the study chose not to exclude participants based on lack of smartphone ownership. Additionally, we chose to exclude a subset of GPS data points to be conservative. If the data transmission between sensor and smartphone occurred more than 10 min after the rescue inhaler event, we disregarded the geolocation because sufficient time had passed to allow movement away from the original location of rescue inhaler use and could misrepresent the location. Lastly, geolocations were not captured if the smartphone was not communicating fully with the sensor (e.g., phone battery expired, Bluetooth turned off, location services deactivated, or insufficient cell or Wi-Fi networks). In these scenarios, all actuations and their date and time data were still collected and stored on the sensor for future transmission, so these data were never lost. Smartphone ownership was the key predictor of GPS data collection; there were no differences in other predictors such as sex, race, smartphone type or time of day.

The geographic data enabled analyses that were not possible previously, such as a kernel density analysis of inhaler use, which highlighted those neighborhoods with the most frequent rescue inhaler use. Additionally, these data also enabled an analysis of the associations of built environment factors with rescue inhaler use. These data could improve upon the limitations of previous studies by identifying nonresidential locations of exposures and even the protective effects of built environment factors.

### Statistical Approach

Negative binomial regression models, including unadjusted and adjusted models, have been used in previous studies to identify asthma exacerbations (Casale et al. 2015). We tested the reliability of our findings from the unadjusted and adjusted zero-truncated negative binomial models against three sensitivity analyses that used different statistical approaches or subsets of the data. In all three sensitivity analyses, we demonstrated consistent and significant findings. AQI, PM_10_, mold, and weed pollen were found to be positively and significantly associated with rescue inhaler, while tree pollen and tree cover demonstrated a negative and significant association.

In our primary analysis, we did not use the individual rescue inhaler use event as the unit of analysis because we needed to control for the differences across individuals in asthma severity and sensitivity to environmental triggers, but did not have these data. Instead, we used the number of active participants per day as an offset to address the impacts of individual user variability. The offset controls for situations when similar exposures between two different days result in dissimilar number of rescue inhaler use events due to differences in participant composition. In our sensitivity analysis using just the subset of the data with demographic information, we could control for variability across individuals using race/ethnicity, sex, smoking status, and pet ownership information and found consistent results. In future studies, we will have access to demographic, socioeconomic, asthma severity, and self-management information on all participants, which will allow us to use the individual rescue inhaler use event as the unit of analysis and control for variability across individuals, which will greatly enhance the utility of these data.

### Consistency with Previous Evidence

The modeling results indicated that data collected by the inhaler sensors have the potential to identify environmental triggers associated with inhaler use. The results presented here are largely consistent with findings from previous studies that demonstrate that environmental triggers such as air pollution, mold, and weed pollen significantly contribute to asthma symptoms. AQI and PM_10_ have been found to be positively and significantly associated with rescue inhaler use (Shadie et al. 2014; Esposito et al. 2014; Brunekreef et al. 2009), as have weed pollen and mold (Toh et al. 2014; Jariwala et al. 2014).

The distribution of vegetation, specifically tree cover, was found to be protective against asthma rescue inhaler use in Louisville. These results add an interesting finding to the literature, given the conflicting results demonstrated in the few existing studies on this topic. In one study, street tree cover was found to be positively associated with asthma and allergic sensitization (Lovasi et al. 2013), but negatively associated with childhood asthma prevalence in another study (Lovasi et al. 2008). More broadly, the benefits of tree cover have been demonstrated in other health and environmental outcomes, such as increased physical activity and social interaction in green spaces, reduced psychophysiological stress and depression, ameliorated noise and air pollution levels, and regulated microclimates (i.e., moderating ambient temperature and urban heat island effects) (Bowler et al. 2010).

We found inconsistent associations for O_3_, with negative associations in some models and positive associations in others. In previous studies, O_3_ has been shown to negatively impact health (Lee et al. 2013; Jerrett et al. 2013; MacIntyre et al. 2011), including asthma (Brandt et al. 2014; Perez et al. 2012). The inconsistent O_3_ results were likely due to insufficient O_3_ sampling data; O_3_ data were only available from three locations throughout Jefferson County at 8-hr intervals. We plan to address this limitation in the next implementation of this study by deploying stationary ozone monitors in specific neighborhoods of interest.

The feasibility study also identified areas for future research into built environment influences on asthma. For example, we determined that public places such as public utilities and railroads may be associated with higher rescue inhaler use. Interestingly, other frequently-used public spaces such as educational sites, religious gathering places, metro government sites, and residential courtyards were also associated with higher inhaler use. These areas represent locations in which people typically spend a significant amount of time, so this relationship requires further exploration when we can assess quantitatively how long people have spent in these locations. To capture a better assessment of such exposures in the expanded study, we are logging participants’ daily locations, with their consent, by recording geolocation information from their smartphones every 2 hr. These data will provide valuable information for characterizing exposure in space–time for each participant, and will allow for more accurate interpretation of the built environment effects. In the future, these findings may offer ideas for specific municipal interventions in these areas, such as controlling weed growth, reducing exposure to air pollution through improved indoor air filtration, and enhancing tree canopy coverage.

### Limitations and Future Directions

This feasibility study had several key limitations. A larger implementation of this study is currently underway, and the lessons learned from this feasibility study have informed the design of that expansion. First, collection of participant information will be more complete in the expansion. The expanded study is requiring participant data collection during enrollment, including race/ethnicity, date of birth, sex, home neighborhood, and census-based income, and educational levels. Other participant characteristics, such as asthma severity, controller medication adherence, and self-management levels, will be collected throughout the study. We can use these data to address the variability in asthma severity and environmental sensitivity across participants. These data will allow us to develop models that include individual rescue inhaler use events as the unit of analysis, which will greatly enhance our dataset.

Second, the expanded study will enroll a larger sample that is more balanced. The expanded study will enroll more than 500 participants in the Louisville area, and aims to achieve balance in participants’ geographic, demographic, and socioeconomic representation to match the composition of Jefferson County.

Third, geographic data collection will be enhanced by expanding the number of participants with smartphones. Recent national surveys indicate that smartphone penetration is increasing, with 64% of all American adults owning a smartphone. Of these smartphone owners, 62% have used their phone to look up health information (Smith 2015). We are also collecting individuals’ home addresses, which will enable us to explore assigning home geolocations to nighttime rescue inhaler use events if location data are missing.

Fourth, we will be able to characterize participants’ exposure signature with much more accuracy, instead of using regional mean statistics for environmental exposures. We will collect snapshots of participants’ locations every 2 hr, only with their consent, to characterize each participant’s exposure in space–time. By being able to characterize the locations in which participants spend their time, we will be able to provide more insight on the impact of built environment factors on rescue inhaler use.

Fifth, we could not identify if a participant was indoors or outdoors, and we did not have indoor air pollution data, so we were limited to addressing outdoor exposures only. In future studies, by taking a more frequent snapshot of location and by asking for self-reported data from participants on perceived triggers and activities, we will begin to identify these differences in exposure signature.

Sixth, next generation sensor technology has improved data collection. The sensor used in this study has since been retired. The current sensor uses Bluetooth LE, a more efficient and low-energy protocol, and has a battery life of > 18 months, which improves the user experience by making it more passive and seamless.

Seventh, we plan to address the limited air pollution monitoring data by deploying stationary pollutant monitors in specific neighborhoods of interest and by exploring mobile monitoring as well. Local advocacy groups in Louisville have already begun testing stationary sensors.

## Conclusions

In summary, our study identified that it is feasible to use electronic sensors to record asthma inhaler use in space and time. The ability to track rescue inhaler use offered a wireless and passive data collection method that was more objective and less burdensome than traditional patient diaries or aggregated utilization data. The study also confirmed that it is feasible to use data collected by the inhaler sensors to investigate the relationships among asthma rescue inhaler use, environmental triggers, and built environment factors. Several environmental triggers were found to be associated with increased inhaler use, such as AQI, PM_10_, weed pollen, and mold. Conversely, tree cover demonstrated protective effects. The lessons learned from this feasibility study have informed the design of the expanded study currently underway in Louisville, and will help investigate these associations further. The application of these new technologies has the potential to improve our understanding of asthma, both for clinical disease management and public health.

## Supplemental Material

(993 KB) PDF
