# Supplementary material for: Feasibility of Deploying Inhaler Sensors to Identify the Impacts of Environmental Triggers and Built Environment Factors on Asthma Short-Acting Bronchodilator Use
Source: Environ Health Perspect. 2016 Jun 24;125(2):254–61. doi: 10.1289/EHP266 (PMC5289907; doi:10.1289/EHP266)
Supplement: (993 KB) PDF [file EHP266.s001.acco.pdf]

Environ Health Perspect
DOI: 10.1289/EHP266

Note to readers with disabilities: *EHP* strives to ensure that all journal content is accessible to all readers. However, some figures and Supplemental Material published in *EHP* articles may not conform to 508 standards due to the complexity of the information being presented. If you need assistance accessing journal content, please contact ehp508@niehs.nih.gov. Our staff will work with you to assess and meet your accessibility needs within 3 working days.

Supplemental Material

Feasibility of Deploying Inhaler Sensors to Identify the Impacts of Environmental Triggers and Built Environment Factors on Asthma Short-Acting Bronchodilator Use

Jason G. Su, Meredith A. Barrett, Kelly Henderson, Olivier Humblet, Ted Smith, James W. Sublett, LaQuandra Nesbitt, Chris Hogg, David Van Sickle, and James L. Sublett

Table of Contents

Table S1: The correlation coefficient matrix of the environmental trigger variables.
Table S2: Estimated effects (and 95% confidence intervals) of environmental triggers in IQR increments on rescue inhaler use for all the actuations through unadjusted and adjusted zero-truncated negative binomial models.
Table S3: Estimated effects (and 95% confidence intervals) of environmental triggers in IQR increments on rescue inhaler use using the subset of data on 80 participants with demographic information using generalized linear mixed models with repeated measures.
Table S4: Estimated effects (and 95% confidence intervals) of environmental triggers in IQR increments on rescue inhaler using the subset of data with geolocation information using unadjusted zero-truncated negative binomial regression models.
Table S5: Associations of rescue inhaler use with built environmental factors in the unadjusted zero-truncated negative binomial models.
Figure S1: A time series plot of daily number of rescue inhaler use events overlaid with daily PM_{10} concentrations. PM_{10} was standardized by its IQR and daily rescue inhaler use events were

transferred into ratios by dividing them by the number of daily active participants. Temporal variations of rescue inhaler use events generally followed temporal trends of PM_{10}.

Figure S2: A time series plot of daily number of rescue inhaler use events overlaid with daily O_3 concentrations. O_3 was standardized by its IQR and daily rescue inhaler use events were transferred into ratios by dividing them by the number of daily active participants. The relationship was more complicated than that of PM_{10}, with associations only being high in summer of 2012.

Figure S3: A time series plot of daily number of rescue inhaler use events overlaid with daily temperature. Temperature was standardized by its IQR and daily rescue inhaler use events were transferred into ratios by dividing it by number of daily active participants. The two generally followed a similar pattern in year 2012 and the associations decreased in year 2013 during a cooler summer.

Figure S4: The scatter plot of temperature vs. rescue inhaler use events. Temperature was standardized by its IQR and daily rescue inhaler use events were transferred into ratios by dividing it by number of daily active participants. The figure indicates that temperature and rescue inhaler use events had a polynomial relationship.

Table S1: The correlation coefficient matrix of the environmental trigger variables.

	NO2	O3	PM10	AQI	PM25	SO2	Grass	Weed	Tree
O3	-0.45								
PM10	0.28	0.38							
AQI	0.28	0.38	1.00						
PM25	0.37	0.13	0.73	0.73					
SO2	0.20	0.20	0.35	0.35	0.40				
Grass	-0.17	0.22	0.10	0.10	-0.01	0.05			
Weed	-0.11	0.10	0.20	0.20	0.05	-0.03	-0.09		
Tree	-0.09	0.08	-0.04	-0.04	-0.06	-0.03	0.03	0.22	
Mold	-0.34	0.32	0.29	0.29	0.04	0.00	0.12	0.35	-0.07

Table S2: Estimated effects (and 95% confidence intervals) of environmental triggers in IQR increments on rescue inhaler use for all the actuations through unadjusted and adjusted zero-truncated negative binomial models.[a]

		Lag 0	Lag 1	Lag 2	Lag 3
Unadjusted Models	NO_2	**1.033 (1.000, 1.066)**	**1.046 (1.014, 1.080)**	**1.035 (1.002, 1.068)**	1.031 (0.999, 1.065)
	O_3	**0.913 (0.878, 0.950)**	**0.928 (0.892, 0.965)**	**0.931 (0.895, 0.968)**	**0.918 (0.883, 0.955)**
	PM_{25}	1.026 (0.990, 1.062)	**1.038 (1.002, 1.075)**	1.026 (0.990, 1.063)	1.020 (0.985, 1.057)
	PM_{10}	**1.204 (1.158, 1.251)**	**1.206 (1.160, 1.254)**	**1.185 (1.140, 1.232)**	**1.154 (1.109, 1.200)**
	AQI	**1.201 (1.156, 1.248)**	**1.204 (1.158, 1.251)**	**1.184 (1.140, 1.231)**	**1.152 (1.108, 1.198)**
	SO_2	1.027 (0.997, 1.057)	1.028 (0.998, 1.058)	0.998 (0.967, 1.028)	0.976 (0.945, 1.007)
	Grass	**0.992 (0.985, 0.998)**	**0.991 (0.984, 0.997)**	**0.988 (0.981, 0.995)**	**0.989 (0.982, 0.996)**
	Weed	**1.005 (1.004, 1.006)**	**1.005 (1.004, 1.006)**	**1.005 (1.004, 1.006)**	**1.005 (1.004, 1.006)**
	Tree	**0.982 (0.977, 0.987)**	**0.987 (0.982, 0.992)**	**0.982 (0.977, 0.987)**	**0.982 (0.977, 0.987)**
	Mold	**1.153 (1.104, 1.203)**	**1.135 (1.086, 1.185)**	**1.165 (1.116, 1.215)**	**1.167 (1.118, 1.216)**
Adjusted Models	NO_2	0.991 (0.955, 1.027)	0.991 (0.956, 1.028)	0.998 (0.962, 1.035)	0.986 (0.951, 1.023)
	O_3	1.026 (0.968, 1.087)	**1.082 (1.021, 1.146)**	**1.073 (1.012, 1.136)**	1.046 (0.987, 1.109)
	PM_{25}	1.008 (0.970, 1.048)	1.014 (0.975, 1.054)	1.018 (0.979, 1.059)	1.015 (0.976, 1.055)
	PM_{10}	**1.054 (1.002, 1.109)**	1.038 (0.987, 1.092)	1.046 (0.994, 1.100)	1.006 (0.956, 1.058)
	AQI	**1.055 (1.003, 1.108)**	1.039 (0.988, 1.092)	1.049 (0.998, 1.103)	1.008 (0.959, 1.060)
	SO_2	**1.042 (1.009, 1.075)**	**1.037 (1.003, 1.071)**	1.020 (0.986, 1.054)	0.983 (0.949, 1.018)
	Grass	**1.012 (1.004, 1.019)**	**1.012 (1.004, 1.019)**	**1.011 (1.003, 1.019)**	**1.012 (1.004, 1.019)**
	Weed	**1.005 (1.004, 1.007)**	**1.006 (1.005, 1.007)**	**1.006 (1.005, 1.007)**	**1.006 (1.005, 1.007)**
	Tree	**0.991 (0.985, 0.998)**	**0.993 (0.987, 0.999)**	**0.995 (0.989, 1.001)**	**0.991 (0.984, 0.997)**
	Mold	0.962 (0.882, 1.043)	**0.904 (0.822, 0.988)**	0.979 (0.900, 1.058)	0.998 (0.921, 1.074)

[a]Weed, tree and grass are daily pollen counts. Bold text indicates significant and positive associations, and bold and red-colored text indicate significant and negative associations. Lag 0, 1, 2 and 3 refer to, respectively, immediate, one-day, two-day and three-day lagged exposures preceding a rescue inhaler use event.

Table S3: Estimated effects (and 95% confidence intervals) of environmental triggers in IQR increments on rescue inhaler use using the subset of data on 80 participants with demographic information using generalized linear mixed models with repeated measures.[a]

	Lag 0	Lag 1	Lag 2	Lag 3
PM25	0.949 (0.877, 1.026)	0.987 (0.912, 1.067)	0.982 (0.908, 1.063)	1.006 (0.930, 1.088)
PM10	**1.116 (1.025, 1.215)**	**1.112 (1.021, 1.211)**	**1.132 (1.040, 1.233)**	**1.106 (1.015, 1.204)**
O3	1.025 (0.938, 1.120)	1.043 (0.954, 1.140)	1.048 (0.959, 1.146)	1.081 (0.989, 1.182)
NO2	0.951 (0.881, 1.025)	0.954 (0.884, 1.029)	0.951 (0.882, 1.026)	0.943 (0.874, 1.017)
SO2	0.930 (0.876, 0.988)	1.009 (0.951, 1.071)	0.945 (0.889, 1.005)	0.948 (0.892, 1.008)
AQI	**1.115 (1.024, 1.214)**	**1.114 (1.024, 1.213)**	**1.133 (1.041, 1.234)**	**1.110 (1.019, 1.208)**
Grass	1.004 (0.998, 1.010)	1.003 (0.997, 1.009)	1.003 (0.997, 1.009)	1.003 (0.997, 1.009)
Weed	**1.008 (1.002, 1.013)**	**1.009 (1.004, 1.014)**	**1.008 (1.003, 1.013)**	**1.009 (1.004, 1.014)**
Tree	**0.9998 (0.999, 1.000)**	**0.9998 (0.999, 1.000)**	**0.999 (0.9989, 1.000)**	**0.9996 (0.999, 1.000)**
Mold	**1.230 (1.111, 1.361)**	**1.164 (1.051, 1.291)**	**1.181 (1.067, 1.309)**	**1.184 (1.069, 1.311)**

[a]Weed, tree and grass are daily pollen counts. Bold text indicates significant and positive associations, and bold and red-colored text indicate significant and negative associations. Lag 0, 1, 2 and 3 refer to immediate, one-day, two-day and three-day lagged exposures preceding a rescue inhaler use event, respectively.

Table S4: Estimated effects (and 95% confidence intervals) of environmental triggers in IQR increments on rescue inhaler using the subset of data with geolocation information using unadjusted zero-truncated negative binomial regression models.[a]

	Lag 0	Lag 1	Lag 2	Lag 3
NO_2	0.966 (0.903, 1.032)	0.951 (0.885, 1.021)	0.946 (0.879, 1.017)	0.914 (0.847, 0.986)
O_3	**1.139 (1.049, 1.237)**	**1.253 (1.149, 1.366)**	**1.201 (1.101, 1.311)**	**1.187 (1.087, 1.295)**
PM_{25}	0.971 (0.899, 1.047)	0.964 (0.889, 1.044)	0.963 (0.887, 1.045)	0.942 (0.866, 1.023)
PM_{10}	**1.224 (1.134, 1.32)**	**1.225 (1.128, 1.328)**	**1.198 (1.101, 1.302)**	**1.120 (1.029, 1.219)**
AQI	**1.225 (1.134, 1.321)**	**1.224 (1.128, 1.328)**	**1.197 (1.100, 1.301)**	**1.122 (1.030, 1.220)**
SO_2	0.999 (0.939, 1.058)	**1.070 (1.011, 1.122)**	1.045 (0.984, 1.100)	1.008 (0.944, 1.069)
Grass	**0.982 (0.967, 0.995)**	**0.982 (0.968, 0.995)**	**0.981 (0.967, 0.994)**	**0.985 (0.971, 0.998)**
Weed	**1.002 (1.000, 1.004)**	**1.003 (1.001, 1.005)**	**1.002 (1.000, 1.004)**	**1.002 (1.000, 1.004)**
Tree	**0.993 (0.982, 1.000)**	1.001 (0.996, 1.005)	**0.992 (0.976, 1.000)**	0.997 (0.988, 1.002)
Mold	**1.302 (1.207, 1.397)**	**1.376 (1.230, 1.538)**	**1.291 (1.191, 1.390)**	**1.271 (1.170, 1.372)**

[a]Weed, tree and grass are daily pollen counts. Bold text indicates significant and positive associations, and bold and red-colored text indicate significant and negative associations. Lag 0, 1, 2 and 3 refer to immediate, one-day, two-day and three-day lagged exposures preceding a rescue inhaler use event, respectively.

Table S5: Associations of rescue inhaler use with built environmental factors in the unadjusted zero-truncated negative binomial models.

		Estimate	Percentile 2.5	Percentile 97.5
Land Use Type	Commercial	1.026	0.973	1.082
	Condominium	0.993	0.985	1.001
	Exempt	**1.153**	**1.113**	**1.194**
	Educational	**1.008**	**1.002**	**1.013**
	Metro Gov't	**1.054**	**1.045**	**1.063**
	Religious	**1.094**	**1.068**	**1.121**
	Condo Master Lot	**1.014**	**1.009**	**1.020**
	Farm	1.003	0.992	1.013
	Utility	**1.009**	**1.005**	**1.013**
	Industrial	**0.992**	**0.987**	**0.996**
	Residential	**0.880**	**0.829**	**0.933**
Land Cover Type	Herbaceous	**0.901**	**0.872**	**0.929**
	Shrubs	0.993	0.955	1.025
	Trees	**0.825**	**0.796**	**0.854**
	All Vegetation	**0.829**	**0.800**	**0.857**

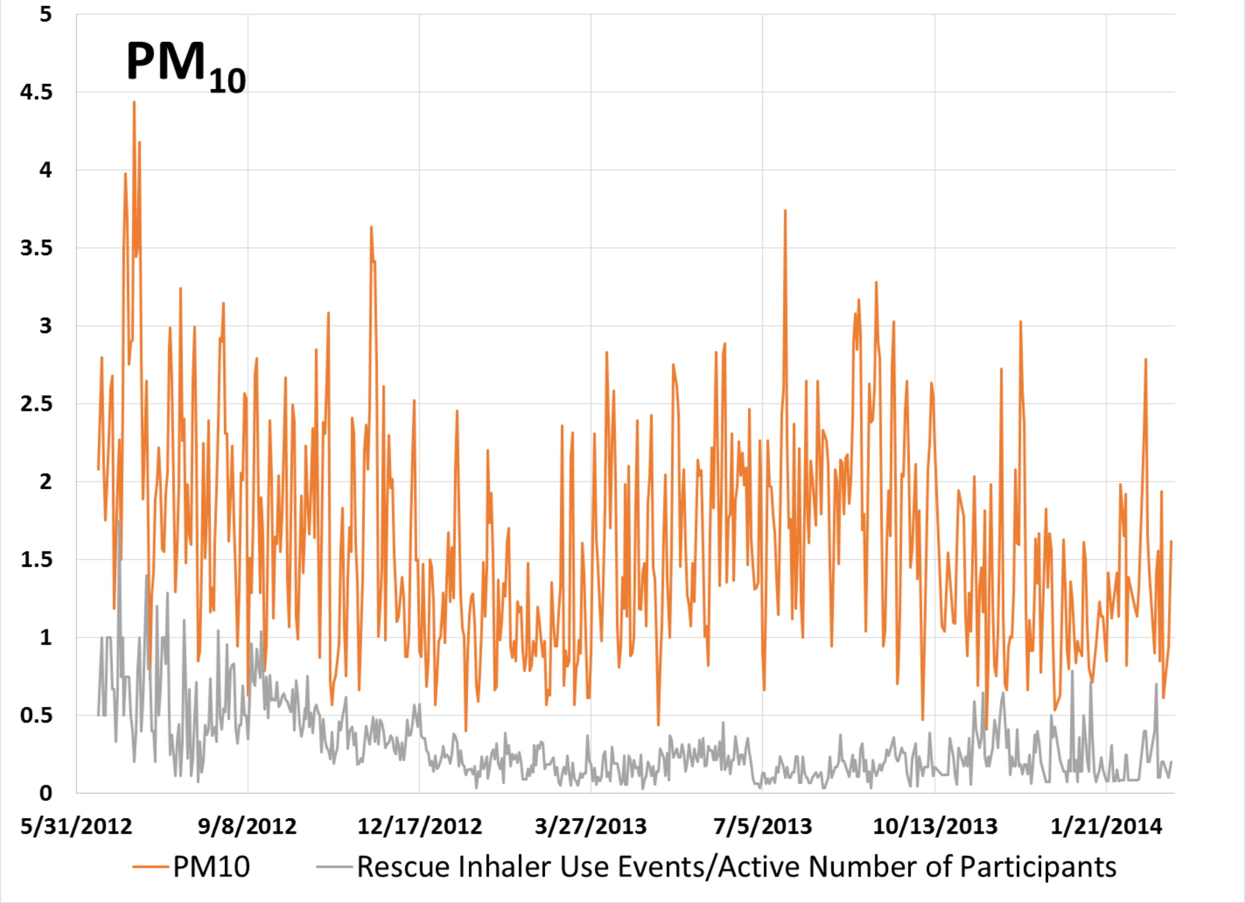

Figure S1: A time series plot of daily number of rescue inhaler use events overlaid with daily PM_{10} concentrations. PM_{10} was standardized by its IQR and daily rescue inhaler use events were transferred into ratios by dividing them by the number of daily active participants. Temporal variations of rescue inhaler use events generally followed temporal trends of PM_{10}.

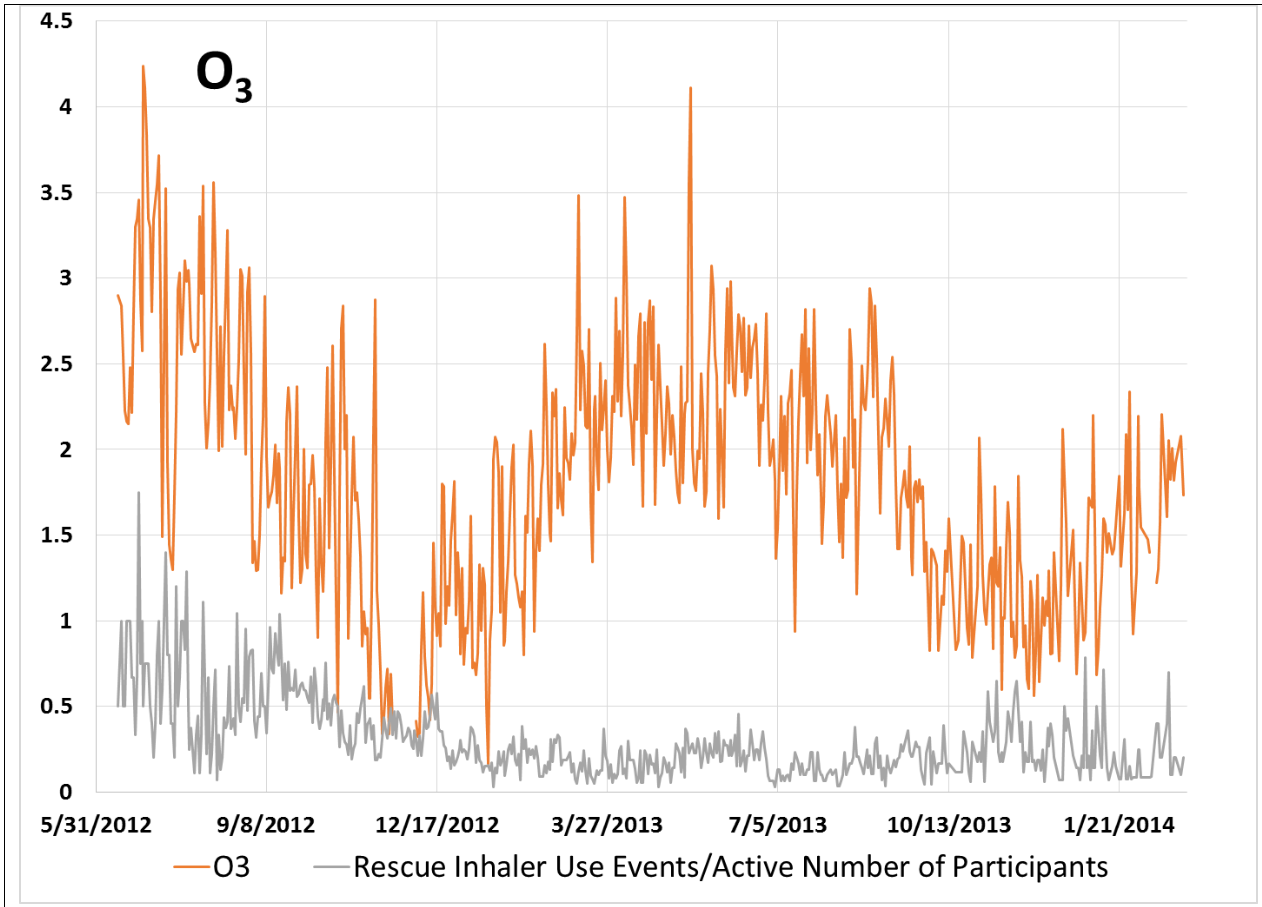

Figure S2: A time series plot of daily number of rescue inhaler use events overlaid with daily O_3 concentrations. O_3 was standardized by its IQR and daily rescue inhaler use events were transferred into ratios by dividing them by the number of daily active participants. The relationship was more complicated than that of PM_{10}, with associations only being high in summer of 2012.

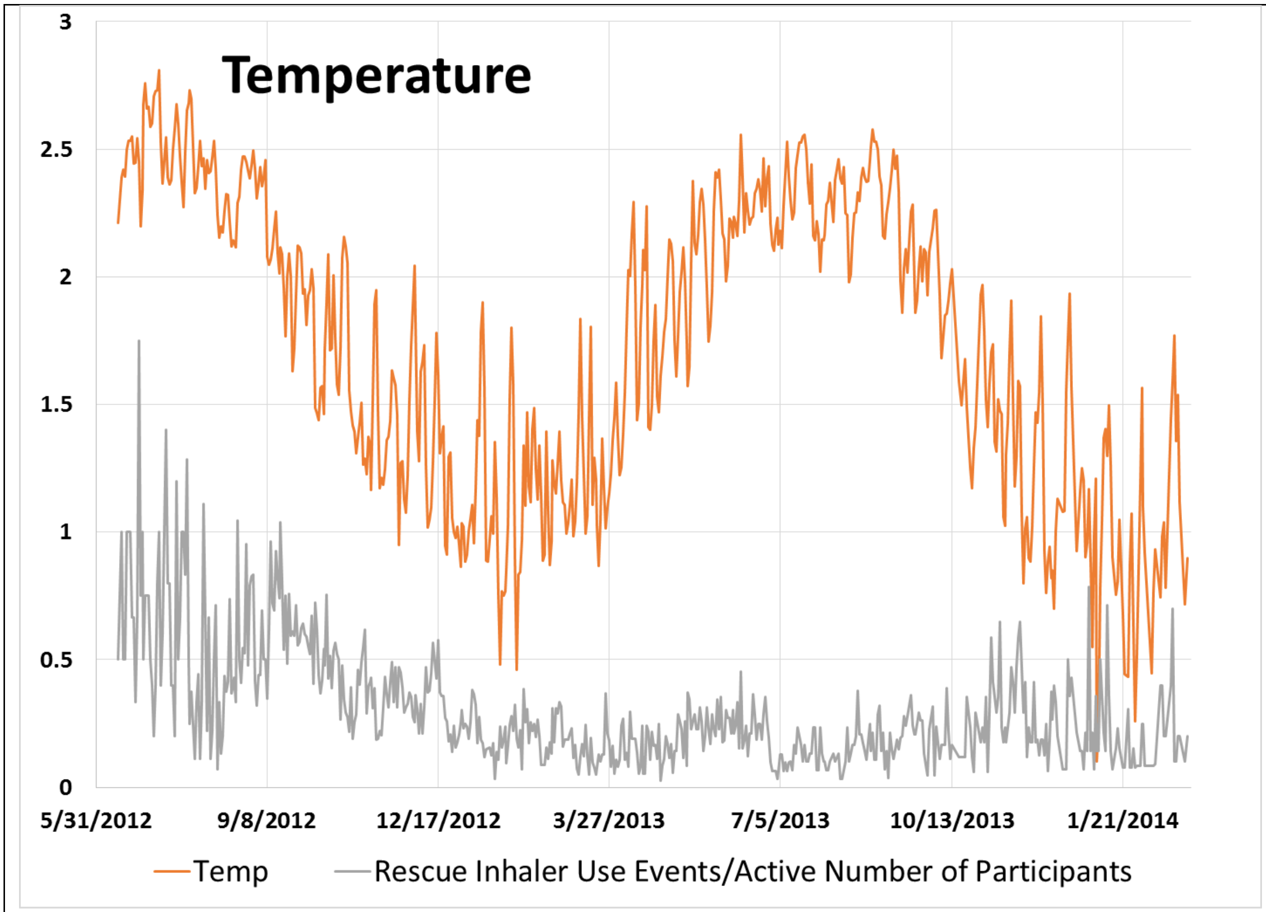

Figure S3: A time series plot of daily number of rescue inhaler use events overlaid with daily temperature. Temperature was standardized by its IQR and daily rescue inhaler use events were transferred into ratios by dividing it by number of daily active participants. The two generally followed a similar pattern in year 2012 and the associations decreased in year 2013 during a cooler summer.

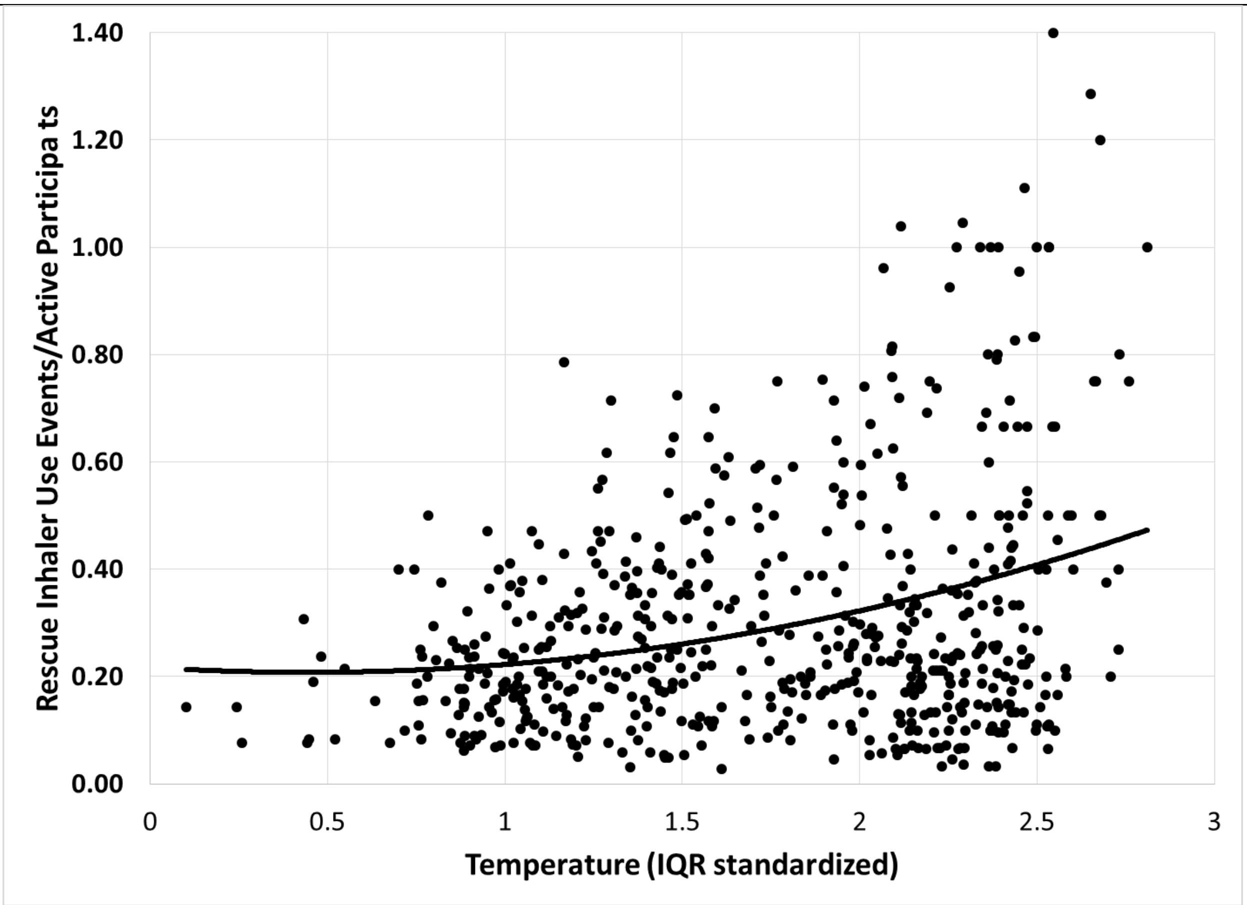

Figure S4: The scatter plot of temperature vs. rescue inhaler use events. Temperature was standardized by its IQR and daily rescue inhaler use events were transferred into ratios by dividing it by number of daily active participants. The figure indicates that temperature and rescue inhaler use events had a polynomial relationship.
